# Real world implementation of blood biomarkers in primary care

**DOI:** 10.1002/alz.090792

**Published:** 2025-01-09

**Authors:** Deanna R Willis, Jared R. Brosch, Nicole R Fowler, Dustin B. Hammers, Diana Summanwar

**Affiliations:** ^1^ Indiana University School of Medicine, Indianapolis, IN USA; ^2^ Indiana Alzheimer's Disease Research Center, Indianapolis, IN USA; ^3^ Indiana Alzheimer Disease Center, Indianapolis, IN USA; ^4^ Regenstrief Institute, Indianapolis, IN USA; ^5^ Indiana University Center for Aging Research, Indianapolis, IN USA

## Abstract

Primary Care (PC) clinicians are faced with numerous competing demands and priorities for maximizing patient care. These challenges make the implementation of strategies for early detection of Alzheimer’s Disease (AD) complex. Few real‐world implementation projects about early detection of AD in PC exist. From 2022‐2023 the Davos Alzheimer’s Collaborative (DAC) Health System Preparedness Flagship project included seven United States academic affiliated PC clinics. We implemented digital cognitive assessments using the Linus Health Core Cognitive Evaluation at routine PC encounters. Patients who failed the cognitive assessment were offered, as part of a research pilot, C2N Diagnostics’ blood biomarker (BBM) test, PrecivityAD, with results disclosure. PCPs were approached to receive education about BBM for AD and to consent to deliver results themselves or defer delivery to the research team. Of the patients approached for BBM, more than half declined. Providers were split on their willingness to deliver results. For patients whose provider did not elect to deliver results or who was unable to deliver results in the timeframe needed, a trained Registered Nurse was trained and observed to disclose results. This session will share perspectives on these real‐life facilitators and barriers to implementing cognitive screening and BBM in PC and how more fully engaging the whole primary care team may help mitigate some of these barriers.

